# Clinical management of portal vein thrombosis in cirrhosis: an evidence‑based narrative review

**DOI:** 10.1186/s42155-026-00671-1

**Published:** 2026-03-18

**Authors:** Kimi Dai, Navjyot Hansi, Teik Choon See

**Affiliations:** 1 Department of Radiology, University of Cambridge, Cambridge, UK; 2 Cambridge Liver Unit, Cambridge University Hospitals NHS Foundation Trust, Cambridge, UK; 3 Department of Radiology, Cambridge University Hospitals NHS Foundation Trust, Cambridge, UK

**Keywords:** Portal vein thrombosis, Cirrhosis, Anticoagulation, TIPS, Thrombolysis, Guidelines, Liver transplantation

## Abstract

Portal vein thrombosis (PVT) complicates up to a third of patients with cirrhosis and is associated with variceal bleeding, refractory ascites, and challenges at liver transplantation. Management has evolved from selective anticoagulation to broader use of endovascular therapies, especially transjugular intrahepatic portosystemic shunt (TIPS) and portal vein recanalisation strategies. In this narrative review, we summarise the current evidence for anticoagulation, thrombolysis, TIPS, and surgery, and compare major society guidelines. Meta-analyses in cirrhosis show that anticoagulation increases recanalisation and reduces thrombus progression without increasing major bleeding and may lower variceal bleeding risk. Endovascular meta-analysis demonstrates high feasibility of TIPS for PVT (~ 95%) with a 12-month portal vein recanalisation around 80% and shunt patency ~ 84% with major complications observed in ~ 10%. Additional catheter-directed thrombolysis during TIPS may increase severe complications and is not routinely recommended in patients with cirrhosis. Guidelines broadly recommend anticoagulation for recent, clinically significant PVT and reserve TIPS for patients with portal hypertension complications, failure of anticoagulation, or transplantation candidacy.

## Introduction

Portal vein thrombosis (PVT) is a frequent vascular complication of cirrhosis that reflects the interplay of stasis from portal hypertension, endothelial injury, and prothrombotic shifts in the cirrhotic haemostatic balance. Clinical consequences include variceal haemorrhage, worsening ascites, portal cholangiopathy, intestinal ischaemia when mesenteric veins are involved, and technical difficulties at liver transplantation (LT) [1, 2]. Recent consensus statements stress standardised nomenclature (recent/acute vs chronic; degree of occlusion; extension to superior mesenteric vein) to guide management [2]. Despite growing evidence, practice varies internationally: some centres favour observation of partial, incidentally detected PVT, whereas others initiate early anticoagulation or offer endovascular therapy when portal hypertension complications coexist [1–5]. This review focuses on adult cirrhotic PVT and synthesises contemporary evidence and guidance for clinicians and interventional teams.

## Search strategy and selection (narrative review)

We performed a targeted literature search of PubMed and Embase (January 2000–September 2025) using combinations of terms including “portal vein thrombosis”, “cirrhosis”, “anticoagulation”, “direct oral anticoagulants”, “TIPS”, “portal vein recanalisation”, “thrombolysis”, and “guidelines”. We prioritised systematic reviews, meta-analyses, practice guidelines (EASL 2016; ISTH 2020; ACG 2020; AASLD 2021; Baveno VII 2022), and large observational cohorts.

## Definitions and classification

Recent (or acute) PVT typically refers to thrombosis detected within ≤ 6 months, often with potentially reversible clot and amenable to anticoagulation and, in selected cases, thrombus-directed interventions. Chronic PVT (> 6 months) is characterised by organised thrombus and/or cavernous transformation. Clinically significant PVT generally denotes ≥ 50% luminal occlusion of the main portal vein and/or extension to the superior mesenteric vein, particularly when symptomatic or when impacting transplant candidacy [1, 2].

### Natural history without treatment

**Fig. 1:**
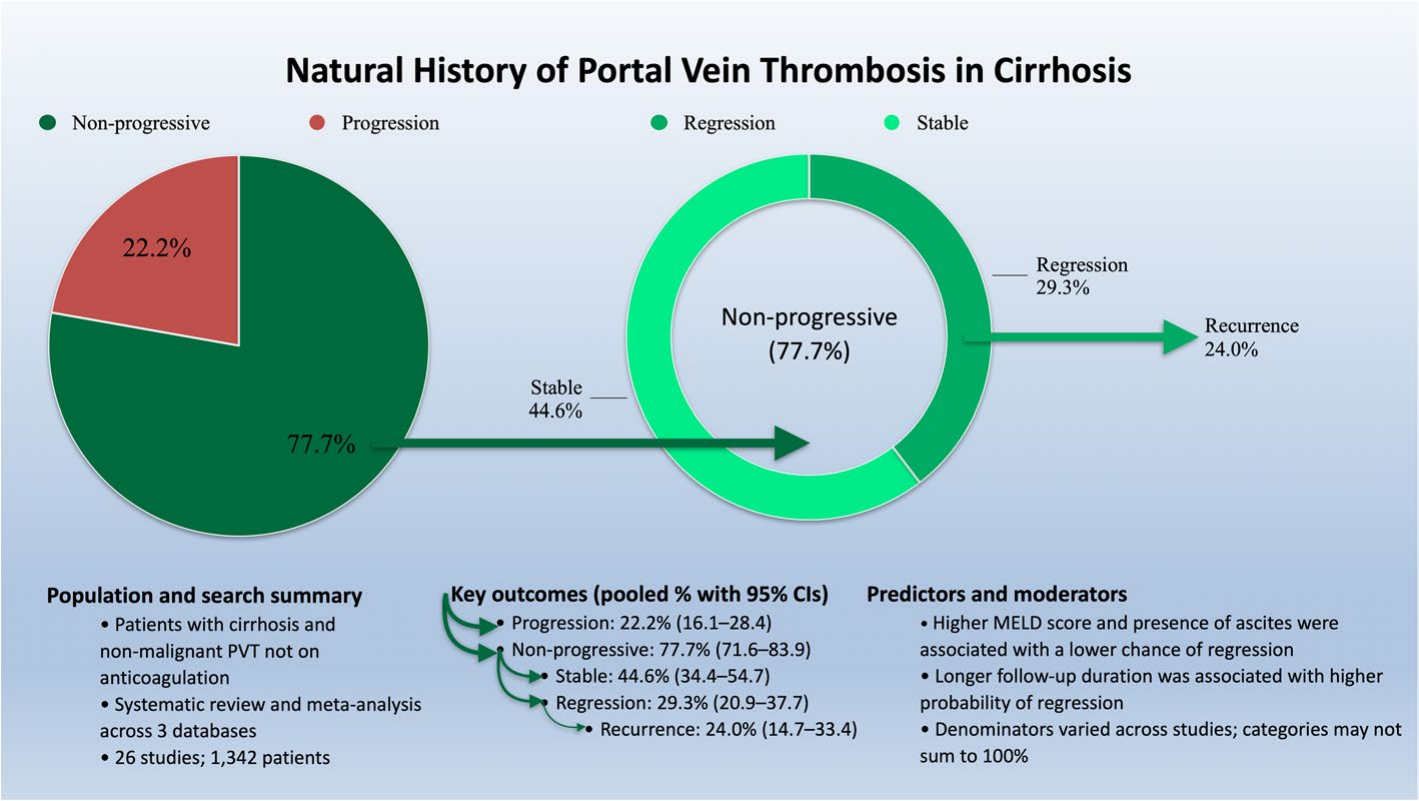
Natural history of cirrhotic PVT, adapted from Giri S., et al. Natural history of portal vein thrombosis in cirrhosis: a systematic review with meta-analysis [9]

Observational data suggest that a proportion of partial, recent PVT regresses or remains stable without anticoagulation. A 2018 review introduced the concept of “transient PVT”, with pooled transient events ~ 40% but marked heterogeneity [8]. A 2023 meta-analysis of cirrhotic PVT reported pooled regression (partial or complete recanalisation) ~ 29% and complete recanalisation ~ 10% under observation, with progression in ~ 22% [9] (Fig. 1). These data underscore the need to individualise therapy based on clot extent, dynamics on interval imaging, portal hypertension complications, and transplant considerations.

### Anticoagulation

#### Rationale and efficacy

Meta‑analysis of eight studies (*n* = 353) showed anticoagulation in cirrhotic PVT increased any recanalisation (71% vs 42%) and complete recanalisation (53% vs 33%) and reduced progression (9% vs 33%) without increasing major or minor bleeding; variceal bleeding was lower with anticoagulation [6], a result of variceal reduction due to improved portal venous flow. Subsequent analyses support these findings and suggest benefits for survival in some cohorts [7].

#### Agents and duration

Low-molecular-weight heparin or vitamin K antagonists have the most historical data; direct oral anticoagulants (DOACs) are increasingly used, with observational meta-analyses suggesting comparable efficacy and favourable safety vs warfarin in carefully selected cirrhotic patients according to Child–Pugh score [15, 16, 18, 19]. Guidelines generally recommend at least 6 months of therapy for recent, clinically significant PVT, with extension in transplant candidates or persistent thrombosis [1–3].

DOAC agent and dose patterns are reported in cirrhotic PVT literature. Although guidelines largely address who to anticoagulate and which Child–Pugh classes can receive a DOAC, several cohort studies and one randomised trial also report the actual regimens used. In a prospective cohort of cirrhosis with chronic PVT, rivaroxaban 20 mg once daily and dabigatran 150 mg twice daily were used (Child–Pugh A for rivaroxaban; B/C for dabigatran). A randomised trial in acute non-neoplastic PVT used rivaroxaban 10 mg twice daily [19]. Real-world series treating PVT have reported apixaban 5 mg twice daily (standard VTE dose) and, in sicker patients, apixaban 2.5 mg twice daily (off-label dose reduction), as well as rivaroxaban 20 mg once daily (OD) and dabigatran 150 mg twice daily (BID) [19]. Across studies, dosing is heterogeneous, and several meta-analyses highlight variability in treatment dose and duration, underscoring the need to individualise by liver function, renal function, bleeding risk, and thrombus burden [16].

However, there are hepatic and renal impairment constraints. Contemporary guidance allows DOACs in Child–Pugh A, and with caution in Child–Pugh B, but not advised in Child–Pugh C. Specifically, the American Gastroenterological Association Clinical Practice Update (AGA CPU) notes: apixaban (A: no adjustment; B: caution; C: not advised), rivaroxaban (A: no adjustment; B/C: not advised), dabigatran (A: no adjustment; B: caution; C: not advised), and edoxaban (A: no adjustment; B/C: not advised); several DOACs are not labelled for CrCl < 30 mL/min [18].

When a DOAC is chosen in cirrhotic PVT, most centres mirror standard VTE doses (e.g., apixaban 5 mg BID or rivaroxaban 20 mg OD; dabigatran 150 mg BID) in Child–Pugh A and select Child–Pugh B patients, reserving reduced apixaban dosing (2.5 mg BID) for frail or high-risk patients (off-label) after multidisciplinary review; avoid DOACs in Child–Pugh C and avoid rivaroxaban in B/C (Table 1).

**Table 1:** Comparison of major DOACs for PVT in cirrhosis

DOAC	Doses reported in cirrhosis/PVT studies	Context
Rivaroxaban	**20 mg OD**; **10 mg BID** (acute PVT) [19]	Chronic PVT cohort (Child–Pugh A for rivaroxaban) and acute PVT RCT [19]; variable durations
Apixaban	**5 mg BID** (standard); **2.5 mg BID** (reduced, off-label in sicker pts)	PVT treatment series (retrospective) [19]; reduced dosing widely used in advanced disease though evidence is limited
Dabigatran	**150 mg BID**	Reported in selected PVT cohorts (Child‑Pugh A and selected B); use caution in Child‑Pugh B; avoid in Child‑Pugh C. Renal clearance requires CrCl check
Edoxaban	Dose variably reported across small series; used after parenteral lead-in in some Japanese cohorts	Sparse PVT-specific dose reporting; follow VTE labelling if considered; avoid in Child–Pugh B/C per AGA CPU [18]

#### Practical points

In almost all patients with recent (< 6 months) PVT, and in particular when it is clinically significant, therapeutic-dose anticoagulation is first-line and is sufficient in the great majority of cases if there is no major contraindication [1–3, 6].

In clinical management, the first step is to confirm recent (< 6 months) PVT without cavernous transformation and define the anatomy and severity (main portal vein vs branch, percentage occlusion, SMV involvement, and symptoms). Patients with clinically significant thrombosis (see Sect. "Definitions and classification" definitions and classification) should receive appropriate varices prophylaxis, but anticoagulation should not be delayed as this reduces the odds of PV recanalisation. Follow-up imaging should also be planned to assess response (at ~ 3 months, and again at ~ 6 months, or sooner if there is clinical change) [1–3, 6, 18].

Observation is reserved only for small (< 50%), incidentally detected thrombi without symptoms, without SMV involvement, and in whom transplant candidacy is not relevant or where bleeding risk is high and outweighs benefits of recanalisation. In this case, we should repeat interval imaging (at ~ 1–3 months) to confirm stability, and start anticoagulation if the clot progresses or extends [1–3, 9].

### Systemic thrombolysis

Across guidelines, thrombolysis is not recommended routinely for cirrhotic PVT given uncertain benefit and bleeding risk. Selective use may be considered in expert centres for acute PVT with mesenteric involvement and clinical deterioration or ongoing intestinal ischaemia despite adequate anticoagulation [2–5].

### Transjugular intrahepatic portosystemic shunt (TIPS)

#### Evidence

A meta-analysis of endovascular therapy for benign PVT (predominantly cirrhotic) reported pooled TIPS feasibility of 95%, 12-month portal vein recanalisation of 79%, and 12-month shunt patency of 84%; major complications were 10% [10]. Additional catheter-directed thrombolysis during TIPS increased severe complications (~ 18% vs 3%) [10]. TIPS also reduces variceal rebleeding in cirrhosis (outside PVT-specific trials) and remains standard, in select patient cohorts, for refractory ascites and variceal haemorrhage.

#### Indications and timing

**Fig. 2:**
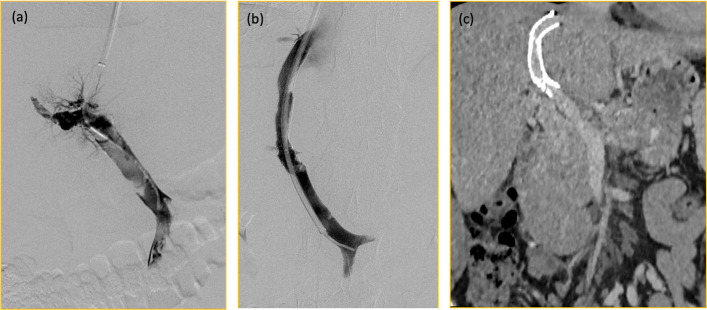
**a** Thrombosed portal vein on portal venography during TIPS. **b** Recanalised portal vein following thrombectomy, thrombosuction, and catheter directed thrombolysis. **c** Patent portal vein, 2 years after TIPS

In cirrhotic PVT, TIPS should be viewed as an exception or rescue strategy rather than a parallel first-line option. It is usually reserved for (a) recent PVT with red-flag features, or with progression of thrombosis despite adequate anticoagulation; (b) chronic/organised thrombosis of the main portal vein that threatens liver transplant feasibility, where portal vein recanalisation with TIPS can restore portal inflow and enable end-to-end anastomosis at LT; or (c) refractory portal-hypertension complications (recurrent variceal bleeding or refractory ascites) despite optimal medical and endoscopic therapy [1–3, 11–14] (Fig. 2).

#### Complications

Hepatic encephalopathy (HE) remains the main trade-off, with contemporary series reporting new or worsened HE in roughly a quarter to one-half of patients over the first year, depending on selection and stent strategy [17]. Clinical factors linked with a higher risk of post-TIPS hepatic encephalopathy are listed in Table 2.

**Table 2:** Clinical factors linked with a higher risk of post-TIPS hepatic encephalopathy

Risk factor	How it can affect decision-making
Pre-existing overt or covert HE	Higher risk of post-TIPS HE. If TIPS is needed, consider prophylaxis, closer follow-up, and a smaller shunt strategy
Poor liver reserve (MELD > 18)	Higher MELD or Child–Pugh class, high bilirubin, and low albumin can raise HE risk and short-term mortality. Use extra caution when MELD > 18; in elective TIPS cohorts, 90-day mortality is ~ 35% with MELD 18–24 and ~ 66% with MELD ≥ 25 (overall ~ 40–45% once MELD ≥ 18) [23]. This can shift the balance away from elective TIPS
Sarcopenia	Low muscle mass can reduce ammonia clearance. Consider nutrition and exercise support, and treat reversible triggers
Renal impairment (and/or hyponatraemia)	Raised creatinine, low sodium, or reduced kidney function can raise HE risk. Optimise fluids, diuretics, and renal function when possible
Older age (caution if > 70 years)	Risk rises with age. Use extra caution if > 70 years, plan shared decisions, and consider closer monitoring after TIPS
Small liver volume or marked atrophy	Lower liver reserve can raise HE risk. Use extra caution in patient selection
Large spontaneous portosystemic shunts	Extra shunting can add to HE risk. Consider shunt embolisation in expert centres when appropriate
Procedure-related shunt effect	Large stent diameter or a very low post-TIPS portal pressure gradient can raise HE risk. Consider 8 mm stent or under-dilation, and avoid over-shunting

Prophylaxis against post‑TIPS hepatic encephalopathy remains an area of evolving evidence. In a multicentre, randomised, double‑blind trial, cirrhotic patients were started on rifaximin 600 mg twice daily 14 days before TIPS and continued for approximately 6 months post-procedure, and demonstrated significantly reduced overt HE compared with placebo (34% vs 53%) [20]. Subsequent reviews and meta‑analyses have reported mixed results for rifaximin monotherapy, with signals that combination regimens (rifaximin plus lactulose) may further lower risk in selected patients, although certainty is limited [21]. Current consensus statements do not mandate routine prophylaxis for all patients; several recommend an individualised approach and note that rifaximin 550 mg twice daily may be considered before non‑urgent TIPS in those with prior observable HE or other high‑risk features [21, 22].

### Combined interventions

Adjunct approaches during portal vein recanalisation and TIPS, such as mechanical thrombectomy or thrombo aspiration, can be used when thrombus burden is extensive or when rapid restoration of portal flow is needed. Case reports and small series describe percutaneous, device-assisted mechanical thrombectomy (for example, AngioJet) that is often combined with catheter-directed thrombolysis and then TIPS to restore and keep portal vein flow in severe portomesenteric thrombosis [24]. In our institution, using a stepwise protocol of low-dose systemic alteplase followed by TIPS-based CDT with mechanical thrombectomy when needed, at least partial recanalisation was achieved in 86% of patients, with complete recanalisation in 50%, major complications in only 9%, and no deaths [25].

**Fig. 3:**
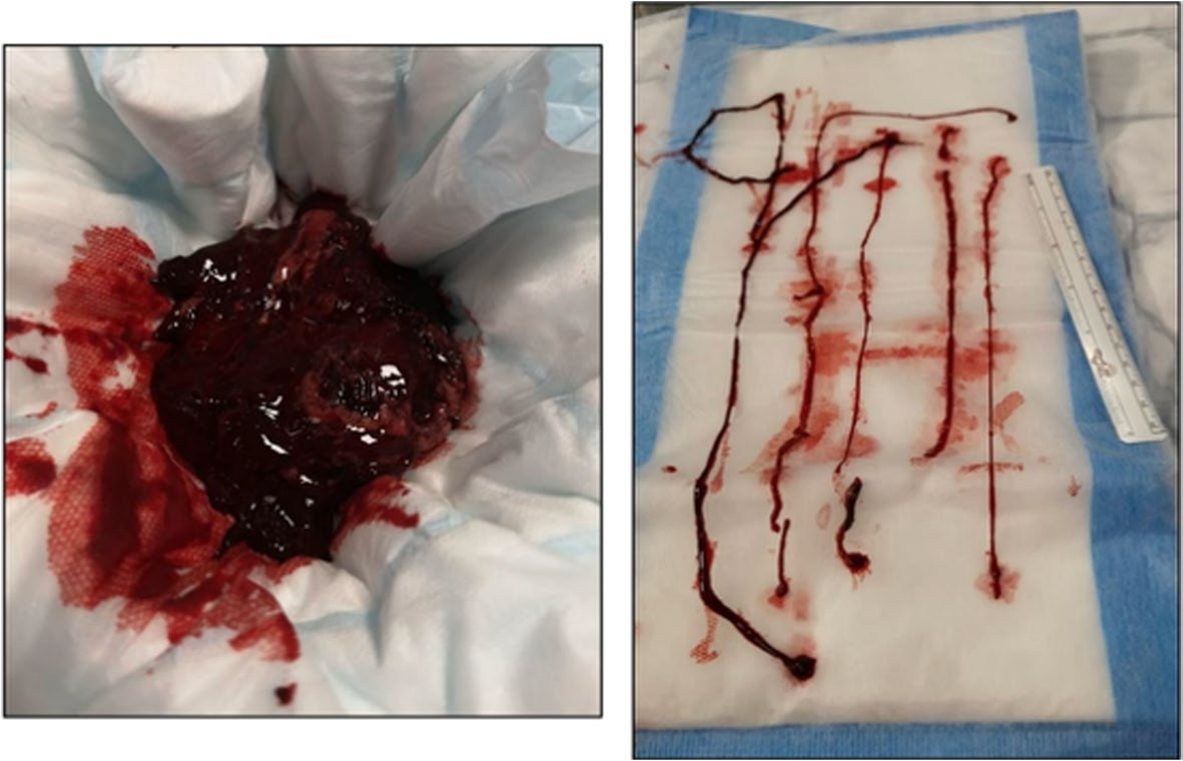
Clot aspirated

For TIPS with catheter-directed thrombolysis (CDT) enhanced outcomes with recanalisation rates as high as 81–100%, at the cost of slightly increased bleeding risk. Meta-analysis showed TIPS + CDT was associated with ~ 17.6% major haemorrhage vs 3.3% with TIPS alone [10]. CDT is useful when thrombus burden is extensive (Fig. 3).

### Surgical options

Outside of transplantation, surgery has a limited role in cirrhotic PVT. Emergent surgery is indicated for bowel infarction due to mesenteric ischaemia. In paediatrics with extrahepatic portal vein obstruction, a meso-Rex bypass is standard; this is rarely applicable to adult cirrhosis. At LT, surgical thrombectomy or non-physiologic inflow procedures may be required when endovascular strategies are not feasible [1].

## Guideline alignment and differences

Baveno VII favours anticoagulation for recent PVT and recommends considering TIPS for refractory portal hypertension complications or when transplant candidacy is affected, with procedures performed in experienced centres. It identifies red-flag features in recent PVT that warrant urgent escalation to expert centres, including persistent severe abdominal pain despite adequate anticoagulation, bloody diarrhoea, rising lactate or lactic acidosis, bowel distention, and second-order superior mesenteric vein branch occlusion. In these cases, early multidisciplinary review and consideration of image-guided recanalisation strategies (including portal vein recanalisation–TIPS) and surgical evaluation are advised [2].

The European Association for the Study of the Liver (EASL) vascular liver disease guideline recommends anticoagulation with low-molecular-weight heparin or vitamin K antagonists for selected cirrhotic PVT cases, particularly when thrombosis is recent or progressive, symptomatic, or relevant to transplant candidacy. Routine thrombolysis is not advised because of limited data and bleeding risk; if undertaken, a transjugular route is noted as the safer access for local therapy. TIPS should be considered when portal hypertension complications persist despite standard care, and it may also facilitate portal access for targeted intervention [4].

The International Society on Thrombosis and Haemostasis (ISTH) guidance emphasises early anticoagulation for acute, symptomatic splanchnic vein thrombosis in the absence of contraindications. Thrombolysis is reserved for highly selected deteriorating patients at expert centres, for example when mesenteric ischaemia is evolving despite adequate anticoagulation. This document focuses on medical therapy and does not provide specific recommendations regarding TIPS [5].

The American College of Gastroenterology (ACG) guideline recommends anticoagulation as first-line therapy for acute PVT in non-cirrhotic patients and for cirrhotic patients when thrombosis is extensive, symptomatic, or involves the mesenteric veins. Thrombolysis may be considered for mesenteric vein thrombosis with impending intestinal ischaemia after failure of anticoagulation. For patients with ongoing portal hypertension complications despite medical and endoscopic therapy, TIPS is appropriate, including its use within transplant pathways when recanalisation is required to enable transplantation [3].

The American Association for the Study of Liver Diseases (AASLD) practice guidance supports anticoagulation for recent, clinically significant PVT in cirrhosis, typically defined by 50 percent or greater luminal occlusion, mesenteric extension, symptoms, or transplant candidacy; small, incidentally detected thrombi without progression can be observed with interval imaging. Thrombolysis should be exceptional, limited to acute cases with persistent intestinal ischaemia despite anticoagulation. TIPS is supported when chronic PVT would otherwise preclude a physiologic portal anastomosis at liver transplantation and for refractory variceal bleeding or ascites not controlled by standard therapy [1] (Table 3).

**Table 3:** Comparison of major guidelines for PVT in cirrhosis

Guideline	Anticoagulation	Thrombolysis	TIPS	Surgery/other
EASL 2016 [4]	Selected cirrhotic PVT; LMWH/VKA; observe small, non‑progressive clots	Not routine; transjugular route preferred if used	For portal hypertension complications; facilitates access for local therapy	Transplant context; otherwise, limited role
ISTH 2020 [5]	Early AC for acute symptomatic SVT; reassess bleeding risk	Only in highly selected deteriorating patients at expert centres	Not addressed	Urgent surgery if intestinal infarction suspected before AC
ACG 2020 [3]	First‑line in acute PVT (non‑cirrhotic) and selected cirrhosis; ≥ 6 mo typical	Consider for MVT with impending ischaemia despite AC	Consider for refractory portal hypertension complications or AC failure; transplant pathway	Limited; transplant setting
AASLD 2021 [1]	Recent, clinically significant or transplant candidates; ≥ 50% occlusion/SMV	Very selective for persistent ischaemia despite AC	TIPS for refractory complications or to enable LT	Transplant thrombectomy/inflow options if endovascular fails
Baveno VII 2022 [2]	Favor AC for recent PVT; monitor small (< 50%) clots	Early image‑guided intervention if red flags of ischaemia	Consider in refractory cases and in LT candidates (expert centres)	Surgery if ischaemia; meso‑Rex in paediatrics (EHPVO)

*EASL *European Association for the Study of the Liver, *ISTH *International Society on Thrombosis and Haemostasis, *ACG *American College of Gastroenterology, *AASLD *American Association for the Study of Liver Diseases, *PVT *portal vein thrombosis, *LMWH *low-molecular-weight heparin, *VKA *vitamin K antagonist, *AC *anticoagulation, *SVT *splanchnic vein thrombosis, *SMV *superior mesenteric vein, *MVT *mesenteric venous thrombosis, *TIPS *transjugular intrahepatic portosystemic shunt, *LT *liver transplantation, *EHPVO *extrahepatic portal vein obstruction

## Practical algorithm

**Fig. 4:**
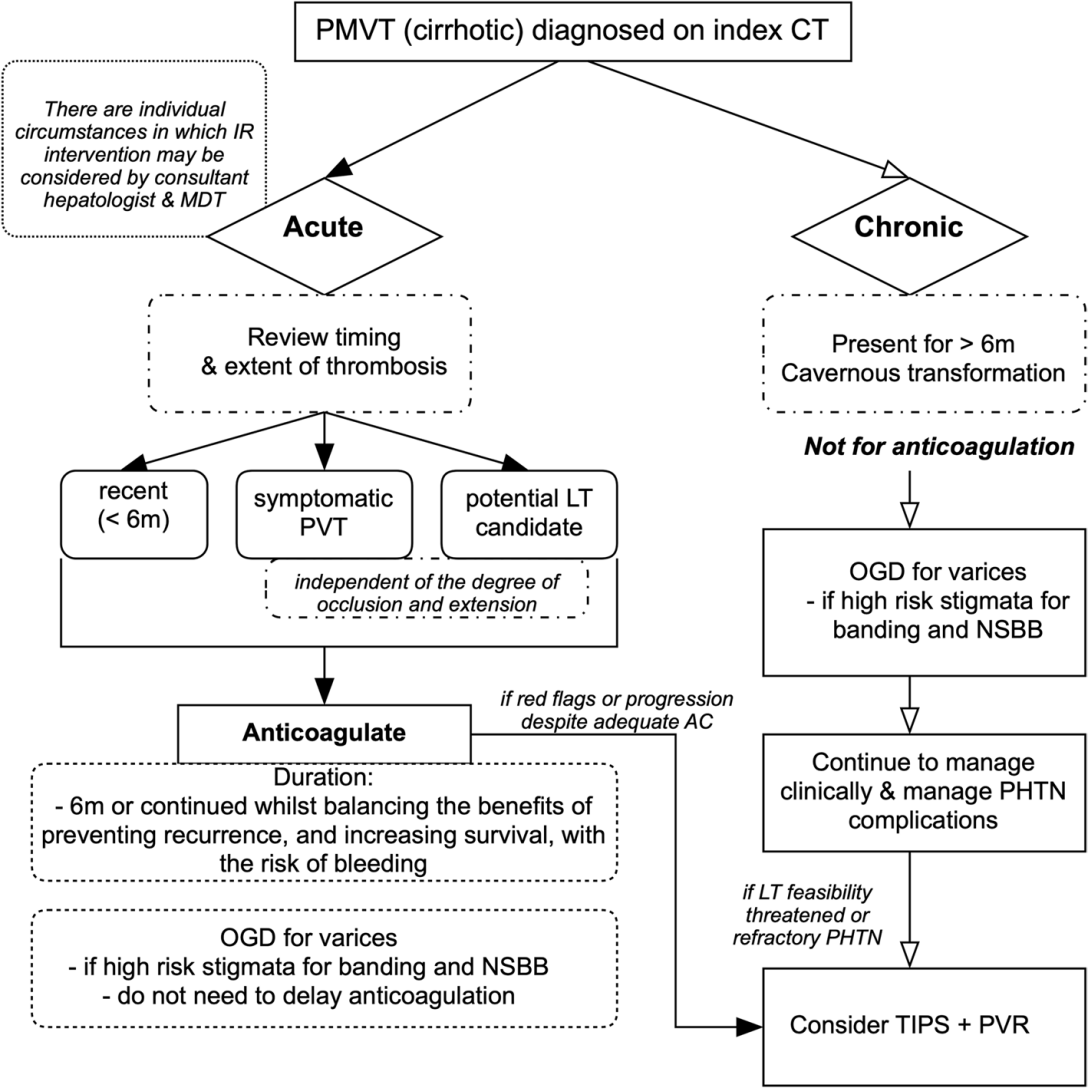
Practical clinical pathway for cirrhotic portal vein thrombosis (PVT)

A practical approach for managing cirrhotic PVT in our institution is summarised in Fig. 4. The guideline is our local adaptation from the major guidelines above. The algorithm distinguishes recent (< 6 months) from chronic (> 6 months) thrombosis, emphasises early anticoagulation for recent, symptomatic, or transplant-relevant PVT, and outlines variceal screening and non-selective beta-blockers where appropriate.

## Conclusions

In cirrhotic PVT, anticoagulation remains the cornerstone pharmacotherapy for recent, clinically significant thrombosis, improving recanalisation and limiting progression without excess major bleeding. TIPS with portal vein recanalisation may be considered when anticoagulation fails in experienced centres. Routine systemic thrombolysis should only be selected with caution in deteriorating acute cases. Treatment should be individualised by clot extent and dynamics, portal hypertension phenotype, bleeding risk, and transplant status, within a multidisciplinary framework. Future research directions include prospective trials of DOACs, refinement of selection criteria for observation vs treatment, and standardised treatment algorithms for PVT.

## Data Availability

Data and material can be acquired upon reasonable request to the corresponding author.
